# Brazilian version of the instrument of environmental assessment* Craig Hospital Inventory of Environmental Factors* (*CHIEF*): translation, cross-cultural adaptation and reliability

**DOI:** 10.1590/bjpt-rbf.2014.0036

**Published:** 2014

**Authors:** Sheyla R. C. Furtado, Rosana F. Sampaio, Daniela V. Vaz, Brena A. S. Pinho, Isabella O. Nascimento, Marisa C. Mancini

**Affiliations:** 1 Physical Therapy Department, Universidade Federal de Minas Gerais (UFMG), Belo Horizonte, MG, Brazil; 2 Physical Therapist, Belo Horizonte, MG, Brazil; 3 Occupational Therapy Department, UFMG, Belo Horizonte, MG, Brazil

**Keywords:** cerebral palsy, questionnaire, environment, social environment, rehabilitation

## Abstract

**Background:**

Environmental factors are essential for the characterization of human functioning and disability; however, the shortage of standardized instruments to assess environmental factors has limited the design of scientific investigations directed at identifying barriers to and facilitators of social participation of people with disabilities.

**Objectives:**

: To translate to Brazilian Portuguese, cross-culturally adapt, and verify the reliability of an environmental assessment questionnaire, entitled *Craig Hospital Inventory of Environmental Factors* (CHIEF).

**Method:**

: The questionnaire was translated to Portuguese, analyzed, translated back to English, and compared with the original version. The final version (CHIEF-BR) was submitted to 47 caregivers of children and adolescents with cerebral palsy (CP). The intra-rater reliability was tested using quadratic kappa and intraclass correlation coefficients (ICC), through interviews of 23 caregivers drawn from the total sample, on two occasions 10 days apart.

**Results:**

: During submission of the questionnaires, it was observed that examples were needed in order to facilitate the understanding of the questions related to the politics sub-scale. Quadratic kappa showed that test-retest reliability of each question varied from 0.28 to 1.0 for the frequency score and from 0.30 to 0.98 for the magnitude score. Intraclass correlation coefficients for total scores showed high consistency indices (ICC≥0.92) for test-retest.

**Conclusion:**

: The Brazilian version of the CHIEF was reproducible and applicable to the study sample. It may serve as an instrument to characterize the environmental barriers as well as a way to document the effects of interventions aimed at minimizing the impact of such barriers on the participation of children and adolescents with CP.

## Introduction

The International Classification of Functioning, Disability, and Health (ICF)^1^, proposed by the World Health Organization (WHO), reflects change in a restricted model of disability for a larger concept, integrating the processes of disability and functionality as part of the health concept and includes, explicitly, the environment as a facilitator or barrier when executing actions and tasks^2^. Participation, which is the involvement of a person in life situations, takes into account the daily experiences of the individual and the context in which he/she performs the activities^1^. Although the WHO recognizes the importance of environmental factors on functioning and disability, lack of standardized instruments to document such factors restricts direct and empirical investigation of barriers and facilitators associated with the social participation of people with disabilities. Existing instruments have a narrow view of the concept of environment, mainly focusing on the architectural barriers and physical aspects^3^ ^,^ ^4^, without apprising the impact of other environmental elements - such as, social support, attitudes, policies, availability of services, among others - that interfere with the components of functionality.

Social participation of children and adolescents with cerebral palsy (CP) has been the subject of interest of several investigators^5^ ^-^ ^10^. Among the factors of interest, several were identified as relevant to social participation for these groups: motor impairment, social support, negative attitude^7^, inclusive actions^6^, and availability of services in the community^9^. Therefore, the profile of functionality of these clinical groups adds specific information about the individuals' health condition, as well as information regarding the environmental factors that may present as barriers or facilitators to social participation of these individuals. It is important to present tools that report these factors, not only to support the process of assessment and clinical intervention but also to support scientific investigations.

The development of a questionnaire called the Craig Hospital Inventory of Environmental Factors (CHIEF) began in 1997. The questionnaire documents the impact of environmental factors on the social participation of people with disabilities^11^. The CHIEF was originally written in the English language and was to be self-administered or administered through interviews with individuals in the age group of 16 to 95 years. It is an instrument consistent with current approaches in the health and rehabilitation areas, such as practice centred on the individual. Such instruments are becoming important resources since they allow the priorities and needs of individuals to be identified and incorporated into the therapeutic approach of health professionals^12^. In recent years, the CHIEF has been widely used to document environmental barriers in different populations^7^ ^,^ ^13^ ^-^ ^17^.

A growing number of translated and adapted instruments have been observed in Brazil due to insightful and scientifically recognized standards in the rehabilitation area^18^ ^-^ ^22^. This process uses specific methodological procedures to translate and assess the need of cross-culturally adapting the instrument, making its practice possible in a new country and language^23^ ^-^ ^25^. In addition, cultural adaptation of an instrument allows for the comparison of results obtained at the international level, which enhances the understanding of a given construct^25^. Therefore, the present study aimed to translate the CHIEF questionnaire into Portuguese and cross-culturally adapt the questionnaire for the people of Brazil. Additionally, a second aim of this study was to determine the test-retest reliability of the questionnaire in a sample of caregivers of children and adolescents with cerebral palsy (CP).

## Method

### Participants

Caregivers of children and adolescents with CP participated in the study. Participants were recruited through contacts with health professionals who work with children and adolescents with CP in hospitals or clinics in Belo Horizonte, MG, Brazil. The inclusion criteria for the children and adolescents were the diagnosis of PC and that they be enrolled in an elementary school. After identifying the participants, their caregivers were contacted. Caregivers who agreed to participate were informed about the objectives of the study and asked to sign an informed consent. If there was a refusal on the part of the caregiver to answer any questions of the CHIEF-BR questionnaire, the participant was excluded from the study. The study was approved by the Ethics Committee in Research of the *Universidade Federal de Minas Gerais* (UFMG), Belo Horizonte, MG, Brazil (protocol nº. ETIC 474/08).

Initially, the demographics of the participants were collected together with the socioeconomic characteristics of the families participating - based on the Brazilian Economic Classification Criterion-ABEP^26^. Children and adolescents with CP were also classified according to the Gross Motor Function Classification System (GMFCS) to identify their levels of gross motor function^27^. This system classified children with a particular emphasis on their walking abilities. Levels I and II represented the child who walked without restrictions; level III was assigned to the child who walked with assistance or support; and levels IV and V differentiated children who used assistive technology to walk from children who, even when using assistive devices, were dependent on others to walk^27^.

### Instrumentation

The CHIEF was developed in two versions: a long and a short version consisting of 25 and 12 questions, respectively^28^. Both versions were subdivided into five domains that were similar to the taxonomy of the environmental factors component of the ICF. These domains were transformed into five subscales and each subscale included the following questions: attitude and support (questions 15, 17, 18, 20 and 21); services and assistance (questions 1, 7, 8, 9, 10, 12 and 14); physical structure (questions 2, 3, 4, 5, 6 and 11); policies (questions 22, 23, 24 and 25), and work and school (questions 13, 16 and 19)^28^.

Each question of the CHIEF was scored according to the frequency of barriers identified (i.e., daily, weekly, monthly, less than monthly, or never) and to the magnitude of barriers (i.e., little problem or big problem). The CHIEF presented three scoring methods for each question: (1) the frequency score, which ranged from 0 to 4, (2) the magnitude score, ranging from 0 to 2, and (3) the score of frequency-magnitude, which was the product of the frequency and the magnitude and varied from 0 to 8. It was possible to calculate the total score of the questionnaire by averaging the scores of frequency, magnitude, and frequency-magnitude of all questions. In this questionnaire, higher score values indicated a greater perception of an environmental barrier.

The psychometric properties of the original version of the CHIEF were examined using a convenience sample of 409 individuals with different diagnoses^14^ ^,^ ^28^. The instrument showed good test-retest reliability of the total score frequency-magnitude (ICC=0.93) and subscales (0.77≥ICC≤0.89). The internal consistency of the total score for frequency-magnitude and subscales, assessed by Cronbach's alpha, ranged from 0.76 and 0.93. The discriminant validity of the instrument was demonstrated by distinguishing individuals with and without disabilities. Although both groups reported having experienced some type of barrier, the group with disabilities reported a higher total mean score of frequency-magnitude and subscales^14.28^.

### Procedure of translation and cross-cultural adaptation

Initially, we contacted the first author of the instrument requesting authorization to translate the CHIEF to Brazilian Portuguese. The translation process of the instrument into the specifics of Brazilian culture and the sample size calculation were based on standard criteria proposed by Beaton et al.^29^. The translation was performed by two independent translators with different backgrounds and whose first language was Brazilian Portuguese. Only one of the translators (T1) was aware of the concepts of the questionnaire. Subsequently, the two translated versions were compared by T1 and by the principal investigator to obtain a single version in Portuguese. The single version was resubmitted for a review to the other translator (T2). Then, an independent translator, whose mother language was English, was blinded with respect to the contents of the questionnaire, and completed the back-translation, producing a new version in English. The version produced by back-translation was compared to the original version of the instrument, with all discrepancies resolved by the translator and the principal investigator. At this stage, it was found that the translated version reflected the same content as the original questionnaire, ensuring consistency of translation. After this stage, the questionnaire was submitted to a committee of experts for consideration.

The committee was composed of three physical therapists, one occupational therapist, and university professors who were familiar with the process of cultural adaptation and were fluent in both languages. The committee prepared a pre-final version in Brazilian Portuguese based on the original questionnaire, the two Brazilian Portuguese translations, the unified translation into Brazilian Portuguese, and the back-translation. In addition to the pre-final version, the expert committee compared the wording of each question from the pre-final Brazilian Portuguese version with the original version in English, focusing on the equivalence of the versions with regard to semantic, idiomatic, and conceptual structure.

The pre-final version of the Brazilian Portuguese questionnaire was then administered to a group of seven caregivers of children and adolescents with CP to ensure comprehension of the questionnaire and the consistency of the procedures. After this step, the pre-final version of the CHIEF was administered individually to 47 caregivers of children and adolescents with CP.

The translated version of the instrument was again administered, by the same investigator, to 23 of the 47 total sample of caregivers who agreed to do the questionnaire again. The second administration of the translated version of the questionnaire approximately ten days after the completion of the first questionnaire had the objective of determining the test-retest reliability. Ten days was considered an adequate amount of time to avoid recall bias and changes in the perception of barriers by caregivers. At the end of the process, the authors of the original version of the instrument were informed in writing of the procedures adopted throughout the study and received a copy of the version of the CHIEF in Brazilian Portuguese, called CHIEF-BR (Appendix 1S *).

### Statistical analysis

Descriptive statistics - central tendency (mean), dispersion (standard deviation), and frequency - were used to characterize the children' and adolescents' age, sex, years of education, and gross motor function, in addition to the socioeconomic status of the families, and the demographics of the respondents of the CHIEF-BR.

The reliability of the instrument was analyzed by two coefficients. The kappa coefficient with quadratic weights (wk) estimated the test-retest agreement on each question, since each question informed, on an ordinal scale, the frequency and magnitude of environmental barrier. For this analysis, the score for frequency and magnitude of each question was used. Secondly, the intraclass correlation coefficient (ICC) examined the test-retest scores of frequency-magnitude of each question; the total scores of frequency, magnitude, and frequency-magnitude of the CHIEF-BR, as well as the mean scores of frequency-magnitude and frequency-magnitude of the subscales since they were considered continuous variables.

Limits for interpretation of the ICC and Kappa used in this study followed the parameters adopted by Gabriel et al.^30^. ICC values greater than a score of 0.75 were considered good indicators of reliability, while values between 0.5 and 0.75 indicated a moderate to good reliability. Values between 0.25 and 0.5 indicated moderate to weak correlation, and values less than 0.25 indicated a very weak correlation.

The parameters used for analyzes of the Kappa index (κ) were as follows: <0.40, poor agreement; κ between 0.40 and 0.60, reasonable or moderate agreement; κ between 0.60 and 0.80, good agreement; and κ above 0.80, excellent agreement^30^ ^,^ ^31^. Analyses were performed using *Stata for Windows* version 10.0 and *Statistical Package for Social Sciences *(SPSS), version 15.0. The level of significance used in the study was set to α=0.05.

## Results

47 caregivers of children and adolescents with CP, who were predominantly female (85%) and had a mean age of 37.81±8.19 years (range: 24-59 years.), participated in this study. Approximately 60% of the participants had completed or partially completed the first grade, 32% had completed the second grade, and 9% had completed the third grade. Most families (66%) of the respondents belonged to economic class C1 and C2, which is equivalent to a monthly income between R$ 726.00 and R$ 1,195.00, according to the Brazilian Economic Classification Criterion ABEP-2008^26^. Descriptive characteristics of the participants (N=47), as well as the subgroup of participants on the test-retest reliability (N=23) are shown in Table 1.

**Table 1:** Descriptive characteristics of the total sample and the subgroup used to test the reliability of the CHIEF-BR questionnaire.

Descriptive variables		Participants	
		Total sample N=47	Reliability Subgroup N=23
Children and adolescents with CP			
Age*	years	9.1±2.5	9.1±2.3
Sex**	F	19 (40.4%)	8 (34.8%)
	M	28 (59.6%)	15 (65.2%)
Formal education*	years	2.70±2.1	2.4±1.7
GMFCS**	I	12 (25.5%)	3 (13.0%)
	II	15 (31.9%)	5 (21.7%)
	III	9 (19.1%)	4 (17.4%)
	IV	7 (14.9%)	7 (30.4%)
	V	4 (8.5%)	4 (17.4%)
**Caregivers**			
Age*	years	37.8±8.2	37.0±8.5
Sex**	F	40 (85.1%)	20 (87.0%)
	M	7 (14.9 %)	3 (13.0%)
Formal education *	years	8.9±3.6	8.1±3.4
CCEB**	A1 and A2	1 (2.1%)	-
	B1 and B2	5 (10.6%)	2 (8.7%)
	C1 and C2	31 (66.0%)	14 (60.9%)
	D	10 (21.2%)	7 (30.4%)

* Numbers indicate mean and standard deviation

** numbers indicate count of children/adolescents and caregivers in the sample and in the subgroup used to investigate the reliability

CCEB = Brazilian Economic classification Criterion (family income categories, in Brazilian Real): A1 and A2= 9.733 to 6.564, B1 and B2 = 3.479 to 2.013, C1 and C2 = 1.195 to 726, D = 485)

sex (F=female; M=male)

- indicates no respondent in this category

**GMFCS:** = Gross Motor Function Classification System.

### Translation and cross-cultural adaptation

During the translation process into Portuguese, some semantic inconsistencies, such as the term *design* and *layout*, which were translated initially by "architecture/design/project", were identified. After a discussion with the translators and the authors of the CHIEF, we opted for the term "physical structure", maintaining the cultural appropriateness of the terminology. This phase allowed the detection of errors and conflicting interpretations of terms with ambiguous meanings in relation to the original version.

During the preparation of the pre-final version by the expert committee, modifications were made to a few words of the unified version. For example, the words "suffered prejudice" were changed to "experienced prejudice", "computer" to "information technology", "obstacle" to "barrier". At this stage, the expert committee had warned that although there were semantic, idiomatic, and conceptual equivalences between the original and the translated versions, it would be important to provide examples during the administration of the questionnaire to facilitate understanding of the questions, especially those related to policies, which could not be understood by people with little education. Faced with this suggestion, the authors of the CHIEF were consulted as to whether or not examples in the body of the questionnaire should be included. However, the authors found it more appropriate to offer examples verbally if the respondent did not understand the question or solicited clarification. The examples were defined prior to administration of the questionnaire and all examiners were instructed to use them when necessary.

The average time required for the administration of the questionnaire was 20 to 30 minutes. During administration, the need for providing examples to facilitate understanding of the questions was observed, as advised by the expert committee. Examples were needed for questions 22, 23, 24, and 25 belonging to the policy subscale (Table 2). After administration of the questionnaire, respondents were asked about the clarity of the questions. Most respondents indicated greater difficulty in understanding the last questions (policy subscale), but such difficulties were eliminated after the examples were provided.

**Table 2:** Examples elaborated to facilitate understanding of the policy questions subscale of the CHIEF-BR questionnaire.

Question	Examples
22	Sports/recreation programs; daycare.
23	Business practices that discriminate against people with disabilities, do not offer support to people with disabilities, do not have adequate accessibility for people with disabilities
24	Rehabilitation programs that pay for the education and equipment necessary for job placement, incentives to employers to hire people with disabilities, private schools that accept students with special needs, education policies that segregate people in special classrooms or schools
25	Laws that protect the rights of the people with disabilities, difficulties to access benefits, loss of some benefits.

### Reliability

The quadratic Kappa indexes of test-retest reliability for each question of the CHIEF were reproducible and ranged from "fair" to "excellent", with the exception of question 23, which showed "poor" agreement (Table 3).

**Table 3:** Classification of the Kappa coefficients obtained for each question of the CHIEF-BR (N=23) in the test-retest reliability assessment for frequency and magnitude scores (should be in title).

Reference criteria for the Kappa coefficient of reliability	Question Frequency score	Question Magnitude score
>0.80 (excellent)	Q2*, Q3*, Q13*, Q14*, Q20*, Q21*	Q2*, Q3*, Q5*, Q10*, Q13*, Q14*, Q17*, Q20*
0.60-0.80 (good)	Q1*, Q4*, Q8*, Q11*, Q12*, Q17*, Q18*, Q19*, Q22*, Q24*, Q25*	Q1*, Q4*, Q6*, Q8*, Q11*, Q15*, Q18*, Q19*, Q21*, Q22*, Q24*, Q25*
0.40-0.60 (fair)	Q5*, Q6*,Q7*,Q9*, Q10*, Q15*, Q16*	Q7*, Q9*, Q12*, Q16*
<0.40 (poor)	Q23	Q23

Q =Question

* =ρ value<0.03.

The ICC of test-retest reliability for the frequency-magnitude scores for each question ranged from 0.29 to 0.98. Most of the questions (48%) showed ICC values above 0.75, indicating reliability between good and excellent. 40% of the questions were of moderate to good reliability, while the remainder of the questions (12%) had poor to moderate reliability (Table 4).

**Table 4:** Classification of the intraclass correlation coefficients obtained for each question of the CHIEF-BR (N=23) in the test-retest reliability assessment for frequency-magnitude scores.

Reference criteria for intraclass correlation coefficient	Question Frequency-magnitude score
>0.75 (good)	Q2*, Q3*, Q11*, Q13*, Q14*, Q19*, Q20*, Q21*, Q22*, Q25*
0.5–0.75 (moderate)	Q1*, Q4*, Q5*, Q6*, Q8*, Q9*, Q10*, Q12*, Q15*, Q17*, Q18*, Q24*
0.25–0.5 (weak)	Q7*, Q16*, Q23
<0.25 (no correlation)	

Q = Question

* =ρ value<0.008.

The ICC of test-retest reliability of the total scores for frequency, magnitude, and frequency-magnitude showed a high level of reproducibility, scoring 0.93, 0.92 and 0.92, respectively. The ICC values of the magnitude-frequency scores of each subscale showed indices of magnitude from moderate to excellent (0.71≤ICC≤0.93). The subscale physical structure showed the greatest magnitude of test-retest reliability, while the policy subscale showed the lowest value. Table 4 shows the ICC of the scores frequency-magnitude scores for each question and Table 5 presents the CCI of the subscales and total scores of frequency, magnitude, and frequency-magnitude.

**Table 5:** Test-retest reliability for the frequency-magnitude scores of each subscale and the total scores of the CHIEF-BR questionnaire (N=23).

Score	ICC	CI (95%)	*p* value
**Frequency-magnitude score **			
Policies	0.71	0.42-0.87	<0.0001*
Physical/Structural	0.93	0.84-0.97	<0.0001*
Work/School	0.89	0.76-0.95	<0.0001*
Attitudes/Support	0.89	0.76-0.95	<0.0001*
Services/Assistance	0.85	0.69-0.94	<0.0001*
**Total**			
Frequency	0.93	0.83-0.97	<0.0001*
Magnitude	0.92	0.82-0.96	<0.0001*
Frequency-Magnitude	0.92	0.83-0.97	<0.0001*

ICC =intraclass correlation coefficient

CI = Confidence interval

* =statistically significant ICC.

## Discussion

This study provides the translated version to Brazilian Portuguese (CHIEF-BR) of a unique instrument in the literature that documents environmental barriers for those with disabilities. Such information is extremely important for understanding the processes of human functioning and disability. The process of translation and back translation was conducted with methodological rigor and in accordance with the standards defined in the literature, achieving conceptual equivalence between the original version and the version in Brazilian Portuguese. Disagreements between the translators and/or the expert committee, when found, were resolved by consensus, prioritizing cultural adaptation to the detriment of semantic equivalence. Changes to any questions of the pre-final version were unnecessary after the administration of the questionnaire to the 47 participants.

During the administration of the CHIEF-BR, it was necessary to provide examples to facilitate respondent understanding. This fact is likely related to the low educational level of most of the sample (60%), that had completed or partially completed primary education. The greatest need for examples was in the policies subscale. However, Kappa indexes for the scores of frequency and magnitude of these questions showed good reproducibility (wk≥0.74), with the exception of question 23, which showed a low rate of weighted kappa for score frequency and magnitude at 0.28 and 0.38, respectively. These results suggest that providing examples appears to be an appropriate strategy to facilitate understanding of the content of the CHIEF-BR and to ensure adequate reproducibility. The low reliability of question 23 may explain the difficulty of the respondents in identifying the impact of policies and rules of corporations dealing with children and young people who have CP.

During the draft stage of the translation of the questionnaire and examples, there were difficulties in distinguishing between the contents of the policy issues subscale. According to the authors of the CHIEF, the perfect distinction between the four areas that make up this subscale, which refers to community, business, education/employment, and the government, is less important than giving the respondents an opportunity to give their perceived ideas about policy barriers for people with disabilities.

The test-retest reliability of this study also showed good levels of agreement for total scores (ICC≥0.92), which were very close to the values reported by the authors of the instrument (ICC≥0.88)^28^. Similarities between the concordance indexes of the CHIEF subscale presented in this study (0.71≤ICC≤0.93) and by authors of the CHIEF (0.77≤ICC≤0.89) were identified, indicating good reproducibility of the instrument even in different contexts.

The authors of the CHIEF compared the concordance of responses provided by individuals with disabilities and their family or friend and it was verified that related parties may not accurately report the barriers faced by individuals (ICC=0.62, measured by the total score of frequency-magnitude)^28^. This result, however, should not be generalized to all populations since it is often difficult to get answers for complex topics, such as perceptions of environmental barriers in the case of children and adolescents with CP. In addition, parents often have a very close relationship with their child or adolescent, and even more so in the case of a deficiency. This close relationship empowers parents, who, in most cases, play the role of primary caregiver, to provide information on the barriers faced by their children, since they also experience the same barriers. We believe that the use of caregivers as respondents of the CHIEF-BR for this population is not only a possible strategy, but is also suitable for gathering information about the environmental barriers imposed on children and adolescents with CP. Most studies that administered the CHIEF to a sample of children whose parents were the respondents did not measure reliability, precluding comparisons of consistency indexes of the instrument at the international level^7^ ^,^ ^15^.

The results of this study indicate that the Brazilian version of the CHIEF (CHIEF-BR), when administered by interview, is appropriate to the population studied and is reliable for measuring the perceptions of caregivers concerning environmental barriers faced by children and adolescents with CP. The test-retest reliability scores of frequency, magnitude, and frequency-magnitude demonstrated good reproducibility. However, it is necessary to investigate the consistency of the instrument in a larger sample. Moreover, it is still necessary to investigate the appropriateness of the CHIEF-BR for other clinical groups which will contribute to its use in Brazil.

We believe that the use of this instrument in a clinical and/or research environment can facilitate an understanding of the processes involved with the functioning and disability of individuals with a range of health conditions. Understanding the environmental barriers can guide local and global actions that are aimed at promoting social participation of individuals assisted by rehabilitation professionals. The CHIEF-BR may also serve to document the effects of interventions aimed at minimizing the impact of environmental barriers to participation of children and adolescents with CP.

## Footnotes

* Supplementary materials are available online at http://www.scielo.br/scielo.php?script=sci_issues&pid=1413-3555&lng=en&nrm=iso

## References

[1] Organização Mundial de Saúde - OMS, Organização Panamerica de Saúde -
OPAS (2003). Classificação internacional de funcionalidade, incapacidade e
saúde.

[2] Nordenfelt L (2003). Action theory, disability and CIF. Disabil Rehabil.

[3] Shumway-Cook A, Patla A, Stewart AL, Ferrucci L, Ciol MA, Guralnik JM (2005). Assessing environmentally determined mobility
disability: self-report versus observed community mobility. J Am Geriatr Soc.

[4] Stark S, Hollingsworth HH, Morgan KA, Gray DB (2007). Development of a measure of receptivity of the physical
environment. Disabil Rehabil.

[5] Vogts N, Mackey AH, Ameratunga S, Stott NS (2010). Parent-perceived barriers to particpation in children
and adolescents with cerebral palsy. J Paediatr Child Health.

[6] Hammal D, Jarvis SN, Colver AF (2004). Participation of children with cerebral palsy is
influenced by where they live. Dev Med Child Neurol.

[7] Law M, Petrenchik T, King GA, Hurley P (2007). Perceived environmental barriers to recreational,
community, and school participation for children and youth with physical
disabilities. Arch Phys Med Rehabil.

[8] Kang LJ, Palisano RJ, King GA, Chiarello LA, Orlin MN, Polansky M (2011). Social participation of youths with cerebral palsy
differed based on their self-perceived competence as a friend. Child Care Health.

[9] Welsh B, Jarvis S, Hammal D, Colver A (2006). How might districts identify local barriers to
participation for children with cerebral palsy?. Public Health.

[10] Kang LJ, Palisano RJ, Orlin MN, Chiarello LA, King GA, Polansky M (2010). Determinants of social participation-with friends and
others who are not family members--for youths with cerebral palsy. Phys Ther.

[11] Harrison-Felix C (2001). Introduction to the craig hospital inventory of environmental
factors.

[12] Law M, Baptist S, Mills J (1995). Client-centred practice: what does it mean and does it
make a difference?. Can J Occup Ther.

[13] Dijkers MP, Yavuzer G, Ergin S, Weitzenkamp D, Whiteneck GG (2002). A tale of two countries: environmental impacts on social
participation after spinal cord injury. Spinal Cord.

[14] Whiteneck GG, Harrison-Felix CL, Mellick DC, Brooks CA, Charlifue SB, Gerhart KA (2004). Quantifying environmental factors: a measure of
physical, attitudinal, service, productivity, and policy barriers. Arch Phys Med Rehabil.

[15] King G, Law M, Hanna S, King S, Hurley P, Rosenbaum P (2006). Predictors of the leisure and recreation participation
of children with physical disabilites: a structural equation modeling
analysis. Child Health Care.

[16] Ephraim PL, Mackenzie EJ, Wegener ST, Dillingham TR, Pezzin LE (2006). Environmental barriers experienced by amputees: the
Craig Hospital inventory of environmental factors-short form. Arch Phys Med Rehabil.

[17] Han CW, Yajima Y, Lee EJ, Nakajima K, Maguro M, Kohzuki M (2005). Validity and utility of the Craig Hospital Inventory of
Environmental Factors for Korean community-dwelling elderly with or without
stroke. Tohoku J Exp Med.

[18] Miyamoto S, Lombardi JI, Berg KO, Ramos LR, Natour J (2004). Brazilian version of the Berg balance
scale. Braz J Med Biol Res.

[19] Mancini MC (2005). Inventário de avaliação pediátrica de incapacidade (PEDI):
manual da versão brasileira adaptada.

[20] Souza AC, Magalhães LC, Teixeira-Salmela LF (2006). Cross-cultural adaptation and analysis of the
psychometric properties in the Brazilian version of the human activity
profile. Cad Saude Publica.

[21] Prado MSS, Magalhães LC, Wilson BN (2009). Cross-cultural adaptation of the developmental
coordination disorder questionnaire for brazilian children. Rev Bras Fisioter.

[22] Amaral M, Paula RL, Drummond A, Dunn L, Mancini MC (2012). Translation of children helping out - responsabilities,
expectations and supports (CHORES) questionnaire into Brazilian-Portuguese:
semantic, idiomatic, conceptual and experiential equivalences and application in
normal children and adolescents and children with cerebral palsy. Rev Bras Fisioter.

[23] Guillemin F, Bombardier C, Beaton D (1993). Cross-cultural adaptation of health-related quality of
life measure: literature review and proposed guidelines. J Clin Epidemiol.

[24] The WHOQOL Group (1995). The Word Health Organization quality of life assessment
(WHOQOL): position paper from The Word Health Organization. Soc Sci Med.

[25] Guillemin F (1995). Cross-cultural adaptation and validation of health
status measures. Scand J Rheumatol.

[26] Associação Brasileira de Empresas de Pesquisa - ABEP (2008). Critério de classificação econômica Brasil 2008.

[27] Palisano RJ, Rosenbaum P, Walter S, Russell DJ, Wood E, Galuppi B (1997). Development and reliability of a system to classify
gross motor function in children with cerebral palsy. Dev Med Child Neurol.

[28] Craig Hospital Research (2001). Craig Hospital inventory of environment factors: version
3.0.

[29] Beaton D, Bombardier C, Guillemin F (2000). Guidelines for the process of cross-cultural adaptation
of self-report measure. Spine (Phila Pa 1976).

[30] Gabriel A Jr, Silva AAB, De Martino MC, Razvickas WJ, Silva RC, Viana AM (2007). Validação do sistema de transporte e das dosagens de
amostras biológicas enviadas para a central de um laboratório de grande
porte. J Bras Patol Med Lab.

[31] Landis J, Koch G (1977). The measurment of observer agreement for categorical
data. Biometrics.

